# How to report and discuss subgroup analyses in clinical practice guidelines? Evaluation procedure of the clinical and statistical relevancy

**DOI:** 10.1007/s10147-025-02774-6

**Published:** 2025-05-11

**Authors:** Kiichiro Ninomiya, Satoru Miura, Yuko Oya, Tomohiro Sakamoto, Kentaro Tanaka, Shunsuke Teraoka, Masahiro Morise, Satoshi Morita

**Affiliations:** 1 Center for Comprehensive Genomic Medicine, Okayama University Hospital, Okayama, Japan; 2 Department of Internal Medicine, Niigata Cancer Center Hospital, Niigata, Japan; 3 Department of Respiratory Medicine and Allergy, Fujita Health University, Toyoake, Japan; 4 Division of Respiratory Medicine and Rheumatology, Department of Multidisciplinary Internal Medicine, Tottori University, Tottori, Japan; 5 Graduate School of Medical Sciences, Research Institute for Diseases of the Chest, Kyushu University, Fukuoka, Japan; 6 Internal Medicine III, Wakayama Medical University, Wakayama, Japan; 7 Department of Respiratory Medicine, Nagoya University Graduate School of Medicine, Nagoya, Japan; 8 Department of Biomedical Statistics and Bioinformatics, Graduate School of Medicine, Kyoto University, Kyoto, Japan

**Keywords:** Subgroup analysis, Guideline, Lung cancer

## Abstract

The results of subgroup analyses of clinical trials are important reference information when considering the generalizability of a study treatment, i.e., providing the best treatment for each individual patient. The results of subgroup analyses are often presented in publications, etc. as forest plots focusing on patient backgrounds. However, it is important to fully understand and grasp some of the issues involved in subgroup analyses and to interpret the results carefully to apply them in clinical practice. Although the literature includes some reports on how subgroup analyses should be evaluated and handled for the purpose of establishing medical practice guidelines, most of the papers have mainly evaluated the reliability of subgroup analyses from a statistical perspective; few of them have incorporated clinical importance in their evaluations. Therefore, in December 2019, we established a Subgroup Analysis Review Committee consisting of oncologists specializing in lung cancer treatment and statistical experts among the members of the Guidelines Review Committee of the Japanese Lung Cancer Association, with the aim of appropriately reflecting subgroup analysis in Japanese lung cancer practice guidelines. We developed a new evaluation strategy to incorporate clinical aspects as well as reliability assessment. Specifically, on the basis of a clinical and statistical review of the problems with subgroup analyses presented as clinical trial results, we developed criteria and procedures to ensure consistency and fairness in the citation of clinical guidelines.

**Supplementary Information:**

The online version contains supplementary material available at 10.1007/s10147-025-02774-6.

## Introduction

Subgroup analysis in clinical trials is a routine procedure in recent randomized controlled trials (RCTs) [1], and forest plots according to patient background factors have been presented to assess the generalizability of the trial results [2]. Examining the treatment effect of each subgroup, such as those based on age [3] and gender [4], which are subgroups of interest for many carcinomas, is informative for decision making in clinical practice, and these results can be an important resource for clinicians in adapting specific treatment options to individual patients [5, 6]. However, the results must be interpreted with caution and with a thorough understanding of some of the biases involved in subgroup analysis. These biases include the possibilities that the group balance of the population guaranteed by randomization may be upset [7], that dividing the population into subgroups reduces power [8, 9], and that the analysis does not consider that patients have multiple characteristics simultaneously affecting the likelihood of benefit [10, 11]. Given these considerations, the results obtained in the subgroup analysis should not be interpreted with undue reliance because they are only an exploratory assessment [9]. However, evaluating the reliability of subgroups is essential to applying subgroup analysis results in practical clinical settings. Nonetheless, an effective strategy to do so has not yet been established. 

How subgroup analyses should be evaluated when considering their reflection in clinical practice guidelines (CPGs) has already been mentioned in several previous reports [10–12]. However, most of these studies have mainly evaluated reliability from a statistical perspective; few of them have incorporated clinical importance into their evaluation. In the present study, we developed a new evaluation algorithm that fully considers both statistical reliability and clinical importance, with the aim of appropriately reflecting subgroup analysis in the Japanese Lung Cancer Society (JLCS) Practice Guidelines. That is, on the basis of a clinical and statistical review of the problems with subgroup analyses presented as the results of clinical trials, we have developed criteria and procedures to ensure consistency and fairness in the citation of clinical guidelines. We have taken care to ensure that the evaluation procedure would be widely applicable not only in our field but also in other clinical oncology fields.

## Motivating example

The programmed cell death 1 ligand 1 (PD-L1), which is expressed on the surface of many cancer cells and immune cells, plays a pivotal role in blocking the cancer–immune cycles by binding the programmed cell death 1 (PD-1). Immunotherapy using PD-1/PD-L1 inhibitors has played a central role in the treatment of not only non–small-cell lung cancer (NSCLC) but also many other types of cancer, bringing about a breakthrough in cancer treatment. The expression of PD-L1 on the tumor cells has been demonstrated to be a useful biomarker for predicting the effectiveness of PD-1/PD-L1 inhibitors in NSCLC treatment. PD-L1 expression is represented as a continuous variable ranging from 0 to 100%. In the KEYNOTE-001 trial, the efficacy of the PD-1 inhibitor pembrolizumab was evaluated in relation to PD-L1 expression [13]. In this trial, a PD-L1 expression level of at least 50% was established as the most effective cutoff line for the efficacy of pembrolizumab. This cutoff line has since been consistently shown in numerous clinical studies to serve as a reliable biomarker for identifying patient populations likely to benefit from PD-1/PD-L1 inhibitors. By contrast, a PD-L1 cutoff of less than 1% is expected to indicate a state where an immune response is either not triggered or is suppressed, suggesting that it could be a negative predictive biomarker. However, the results in this subgroup have been inconsistent and the significance of this biomarker is a topic of ongoing debate.

The PACIFIC trial was a phase III study of durvalumab in unresectable stage III NSCLC after definitive chemoradiotherapy [14]. In this previous study, the hazard ratio (HR) for overall survival (OS) in the subgroup with tumor PD-L1 expression less than 1% was 1.36 (95% confidence interval [CI]: 0.79–2.34), which was inferior to the OS-HR of 0.53 (95% CI: 0.36–0.77) in the at-least-1% subgroup. On the basis of this result, the European Medicines Agency (EMA) has approved maintenance durvalumab only for patients with PD-L1 expression of at least 1% [15, 16]. However, this subgroup analysis had many limitations and biases. First, this post hoc analysis was performed in response to a request by the EMA. Second, the balance of background factors in the subgroup with PD-L1 expression of less than 1% was significantly disrupted because PD-L1 evaluation was not mandatory in the PACIFIC trial. As illustrated by this example, careful evaluation is needed to determine whether biomarkers used in clinical research subgroups should be incorporated into CPGs as important factors for treatment selection.

## Methods

### Processes and targets

This evaluation algorithm was reviewed and developed by a task force consisting of oncologists specializing in lung cancer treatment and statistical experts among the members of the Guidelines Committee of the JLCS. Past reports on the reliability of subgroup analyses were reviewed to identify the necessary domains for evaluating subgroup reliability. Researchers subsequently developed an algorithm by considering the importance of these domains [10–12]. In addition, because the goal of this algorithm is to determine the applicability to guidelines, clinical significance was also incorporated into the algorithm. To evaluate the practical applicability and validity of the developed evaluation procedures, we used as a case study the subgroup analyses of RCTs adopted and discussed in previous JLCS Practice Guidelines [17, 18].

### Elements of subgroup analysis

Subgroup analyses have often been evaluated using “efficacy,” which is shown as a forest plot in RCTs, as the main indicator [2]. When prognosis and health status differ substantially among subgroups, absolute measures such as median values may not be adequate for comparison [12]. One of the principles in assessing the evaluation of subgroup analysis is to use relative measures such as HRs. Therefore, for assessments of progression-free survival (PFS), disease-free survival (DFS), and OS, the HRs and their CIs for survival analyses of RCTs with some assurance of power were used in this algorithm. Clinical trial results for which statistical interactions could not be evaluated and statements from single-arm studies were excluded from this evaluation. As noted in the Discussion section, we do not account for the safety results as an element included in the evaluation algorithm.

### Statistical analyses

In the present study, we carried out interaction tests to evaluate whether group differences exist in treatment effects among the subgroups. However, subgroup analyses in RCTs have often not been subjected to appropriate statistical validation using interaction tests [1]. If the results of the interaction test were reported in the paper in the subgroup analysis under consideration, we adopted the value; otherwise, we approximated the *p* value using Cochran’s *Q*-test [19] from the point estimates and 95% CIs presented for each subgroup. In the present work, statistical analyses were performed using STATA/SE ver. 18.0 (StataCorp LLC, College Station, TX, USA).

## Results

### Evaluation algorism for adapting subgroup analysis to CPGs

**Fig. 1:**
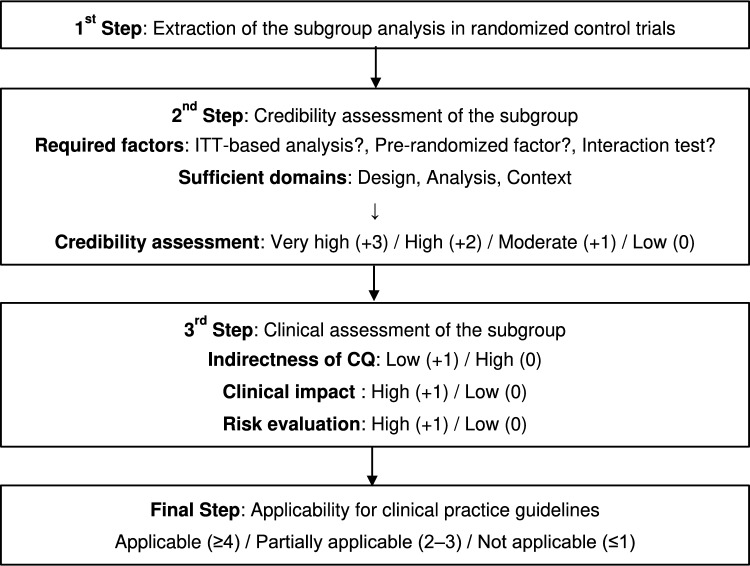
Evaluation algorithm for adapting subgroup analysis to clinical practice guidelines

An overview of the evaluation algorithm to be followed to consider subgroups for inclusion in the CPGs is shown in Fig. 1. This evaluation algorithm comprises three steps: extracting the subgroup analysis in RCTs (step 1), assessing credibility (step 2), and conducting a clinical assessment (step 3). Details of each step are described as follows.

### Step 1: Extraction of the subgroup analysis in RCTs

The first step (step 1) is to identify the reasons for extracting a specific subgroup to reveal any biases that need to be considered in subsequent steps. Subgroups of interest are often selected from either a statistical or a clinical perspective. The subgroup extracted from a statistical perspective is defined as a subgroup that shows a substantial between-group difference in the forest plot obtained from an RCT [2]. These subgroups include those that have also not been routinely included in other clinical trials, such as the interracial subgroups [20] and the metastatic site subgroups [21], and that should be evaluated in the subsequent steps, with particular attention focused on multiplicity and clinical importance. However, the subgroups extracted from the clinical perspective are defined as the analysis of high clinical interest; they have been suggested by the members of the JLCS Guideline Committee or by the public in this algorism. These clinical perspectives have often referred to subgroups already recognized as important in similar previous studies, such as the older subgroup in combination therapy with angiogenesis inhibitors [22, 23] or subgroups based on PD-L1 expression in immune checkpoint inhibitors [14, 24]. These subgroup analyses from a clinical perspective are often proposed by the investigator or the pharmaceutical company. Therefore, the clinical significance and statistical aspects of these subgroups should be carefully re-evaluated after feedback has been obtained from outside parties via public comments.

### Step 2: Credibility assessment of the subgroup

#### Three required factors

The next step (step 2) is to assess the credibility of the subgroup analyses (Table 1A). The credibility assessments were based on the following questions: (1) Does the primary outcome of the study show the clinically meaningful benefit based on the intention-to-treat (ITT) analysis? (2) Was the subgroup variable a pre-randomized factor? (3) Was there a meaningful difference between the subgroups in the interaction test? Among these questions, questions (1) and (2) are primarily intended to evaluate the multiplicity problem of increasing alpha error, whereas question (3) is primarily intended to evaluate the basic premise of whether the multiplicity problem should be discussed as a subgroup analysis. Meeting all three of these criteria is essential to ensure the reliability of a subgroup. If any one of these criteria is not met, the subgroup should not be adopted into the Clinical Practice Guidelines (CPGs) in principle.

**Table 1:** Components of evaluation algorithm of subgroup analysis for clinical practice guidelines

A. Required factors
1. Did the primary outcome of the study show the meaningful benefit based on the intention-to-treat analysis?
2. Was the subgroup variable a pre-randomized factor?
3. Was there a meaningful difference between the subgroups in the interaction test? (*p* < 0.05: surely; 0.05 ≤ *p* < 0.15: plausible)
B. Credibility assessment
Design
1. Was the subgroup hypothesis predetermined?
2. Was the subgroup factor a stratified allocation factor or adjusted for important prognostic factors?
Analysis
1. Was the subgroup analysis performed on the basis of Intention-to-treat?
2. Was the subgroup analysis consistent with the relevant outcomes in this trial?
Context
1. Was the subgroup analysis consistent with other independent clinical trials?
2. Was there a basic research context that could explain the obtained subgroup effects?
C. Clinical assessment
Indirectness of CQ
Is the subgroup non-directive to the assumed clinical question?
Clinical impact
Does the subgroup have a greater clinical impact on treatment decisions?
Risk evaluation
Does the subgroup influence treatment decisions due to risk of harm?

1. Does the primary outcome of the study show the clinically meaningful benefit based on the ITT analysis?

The facts that the primary endpoint of the clinical trial is based on an ITT analysis and that the trial is statistically positive are the most important factors in assessing the reliability of the clinical trial before evaluating the subgroup analysis. Caution should be exercised when cases are excluded from the analysis for reasons such as ineligibility because arbitrary case selection (referred to as attribution bias) may have occurred [25]. In addition, demonstrating usefulness on the basis of subgroup analyses of studies that did not meet the primary outcome is not acceptable from the perspective of elevated alpha error [26]. In view of the above, subgroup analyses of studies in which the main analysis was not performed by ITT analysis or in which the primary outcomes were not statistically fulfilled should be avoided to adapt the CPGs because such analyses are unreliable.

(2) Was the subgroup variable a pre-randomized factor?

It is necessary to consider whether the subgroup factors under analysis were established prior to randomization. For example, analyses based on factors observed after randomization, such as subgroup analyses limited to cases of response to treatment, are not appropriate for subgroup analyses adapted to CPGs because they are likely to have selection bias due to the influence of the study treatment; they are also likely to have multiplicity [27].

(3) Was there a meaningful difference between the subgroups in the interaction test?

Here, the statistical evaluation of group differences in treatment effects among subgroups, which are called subgroup effects, is performed using interaction tests [19]. Because subgroup analyses are potentially underpowered, doubts remain as to whether the analyses are based on mere chance or true events. Therefore, an interaction test should be conducted to evaluate whether further investigation should proceed [8]. In this evaluation procedure, a test result of *p* < 0.05 is defined as “surely” and a test result of 0.05 ≤ *p* < 0.15 is classified as “plausible”; if the test result is* p* < 0.15, we proceed to the next domain evaluation [8, 12]. Even if a large difference is observed in the interaction test, the possibility of chance due to multiplicity cannot be fully ruled out; thus, the evaluation domains that follow must also be judged comprehensively.

#### Three key sufficient domains to assess in evaluating reliability

If all three of the aforementioned required factors are met, the subgroup analysis is then evaluated at three levels (Yes/No/Not evaluable) in the three key sufficient domains of “Design,” “Analysis,” and “Context” (Table 1B). These domains were remade to be clinically tractable with reference to a previous report [12].

### Design

1. Was the subgroup hypothesis predetermined?
2. Was the subgroup factor a stratified allocation factor?/Was the subgroup factor adjusted for important prognostic factors?

If such subgroup analyses were preplanned as part of the study design, the results would be highly reliable because they were validated on the basis of the hypothesis. However, from the viewpoint of alpha error control, it is advisable to confirm in the protocol that only meaningful subgroup factors are preplanned. If the subgroup is a stratification factor, it is more reliable for validating the subgroup effect because the balance of patient backgrounds across treatment groups can be expected to be consistent [7, 10]. Even if the subgroup is not a stratification factor, its reliability may be higher if it is adjusted for other influencing factors (e.g., multivariate analysis).

### Analysis

1. Was the subgroup analysis performed on the basis of an ITT–based population?
2. Was the subgroup effect consistent across the relevant outcomes in the same trial?

To assess directness, it is necessary to confirm again whether ITT analysis is used in the subgroup analysis. In some cases, analyses have included a large number of exclusions from the ITT population because a subgroup factor (i.e., PD-L1 expression) has been unmeasurable [14]; in such cases, reliability should be assessed lower. If inconsistencies are observed for different validity outcomes within the same study, the reliability is evaluated as low. For example, PFS may have been beneficial in a subgroup, but OS benefit may have been lost or disadvantageous in that subgroup [28]. In this case, background biases related to important prognostic factors and the effect of posttreatment and survival after disease progression should be considered in the interpretation of the OS results.

### Context

1. Was the subgroup analysis consistent with other independent clinical trials?
2. Was there a basic research context that could explain the obtained subgroup effects?

Reliability is enhanced from the perspective of reproducibility if the results are found to be consistent with the results of another independent, similar clinical trial in the subgroup factors. Reliability is also enhanced if the causes leading to the results of the subgroup analysis have been identified in the pre-clinical research. However, it is necessary to verify whether the pre-clinical research was reported after the results of the subgroup analysis were obtained. For subsequent pre-clinical research results, it is important to consider the potential for lower reliability.

#### Credibility assessment for subgroup analysis

Finally, the overall credibility of the subgroup analysis is assessed on a four-point scale (Very high, High, moderate, low) on the basis of the three aforementioned domains. Each domain may contain cases that are impossible to evaluate because the results are not shown. If the domains are difficult to evaluate, the assessment is not evaluable (NE). If there is more than one “Yes” in each of the three domains, the reliability is considered to be the highest (Very high); if there are two “Yeses” in the different domains, the reliability is considered to be High; if there is one Yes for only one domain, the reliability is considered to be moderate; and if there is no “Yes,” the reliability is considered to be Low. If the credibility of the subgroup analysis is Low or NE, we do not proceed to step 3.

### Step 3: Comprehensive clinical assessment

Finally, in step 3, the following three clinical assessments are evaluated in addition to the credibility assessment in step 2. (1) Is the subgroup non-directive to the assumed clinical question (CQ)? (indirectness of CQ) (2) Does the subgroup have a greater clinical impact on treatment decisions? (clinical impact) (3) Does the subgroup influence the treatment decisions due to risk of harm? (risk evaluation).

Each of these assessments will be evaluated with a score (+ 1), and the conformity to the clinical practice guideline will be verified. Because these evaluations are expected to differ depending on the subjectivity and values of each evaluator, it is desirable to hold discussions at a meeting of experts composed of members from multiple professions to reach a consensus.

1. Indirectness of CQ: Is the subgroup non-directive to the assumed CQ?

This evaluation procedure is intended to be adapted to practice guidelines. Using the PICO (Patient, Intervention, Control, Outcome) strategy [29], the subgroups being assessed will be evaluated for indirectness to CQ. If the results of the extracted subgroup analyses are consistent with CQs already specified in the guidelines, the integrated evaluation of outcomes will be easier and more important. This item is evaluated at two levels depending on the non-directiveness of the CQ: Low if there is some degree of agreement, and High if there is no agreement.

(2) Clinical impact: Does the subgroup have a greater clinical impact on treatment decisions?

This section evaluates the strength of the influence of subgroup efficacy results on treatment decisions. Clinical impact should be evaluated comprehensively including not only the main efficacy (HR) but also other efficacy outcomes and patient-reported outcomes for each subgroup. If additional information is needed to determine subgroups, such as genetic testing or tumor PD-L1 expression, the simplicity and feasibility of conducting such examinations should be considered. If subgroup analysis is based on information not usually retrieved clinically, the evaluation will be lower.

(3) Risk evaluation: Does the subgroup influence the treatment decisions due to risk of harm?

In assessing the clinical significance of subgroups, it is also important to evaluate the risks, including adverse events, of treatment. If there is a difference in adverse events for each treatment and one treatment has already been shown to be more harmful than the other, the clinical significance of dividing patients into subgroups in that treatment is assumed to increase. Risk evaluation will vary depending on the patient population and the expected degree of efficacy. For example, in the case of perioperative treatment, patients eligible for adjuvant therapy include a subset of patients who are expected to be cured without treatment. For a subgroup of patients with early-stage cancer at low risk of recurrence, interventional treatment itself with adverse events may pose a higher risk. Therefore, for this subgroup, long-term management of adverse events must also be fully considered. However, most clinical trials do not present adverse events by subgroup. Therefore, predictions must be made on the basis of the results of adverse events in the overall population and information such as patient background.

### Final decision

On the basis of the total points calculated in the “Credibility assessment” and “Clinical assessment,” the final evaluation is carried out as follows: four or more points are considered applicable, two or three points are considered partially applicable, and one point or less is considered not applicable. If the subgroup is evaluated as applicable, it is considered to have a strong impact on clinical practice and decision making and should be considered for adoption as a CQ in CPGs. However, if the subgroup is rated partially applicable, it may have an effect on clinical practice but is not essential data and is considered to be only for reference. If the subgroup is rated low, the effect of the relevant subgroup data on the actual clinical practice is considered to be low or the analysis is not statistically sufficient; it should therefore not be adopted into the CPGs.

### Validation of clinical trials

On the basis of this evaluation algorithm, we present actual evaluation results for each domain with two specific examples.

[Case study 1]: Tumor PD-L1 expression subgroups in the PACIFIC trial (Supplementary Table 1 and Fig. 1).

The PACIFIC trial was a phase III study to evaluate the efficacy of durvalumab in unresectable stage III NSCLC after chemoradiotherapy [13]. We discussed whether the guideline should adopt an efficacy evaluation by subgroup of tumor PD-L1 expression greater than or less than 1% in this trial.

First, the co-primary endpoints of PFS and OS were both significantly prolonged with durvalumab. Subgroup analyses based on tumor PD-L1 expression prior to treatment were preplanned with a cutoff value of 25%. On the other hand, the OS-HR for PD-L1 < 1% was 1.36 (95% CI: 0.79–2.34), which was inferior to the OS-HR for PD-L1 ≥ 1% of 0.53 (95% CI: 0.36–0.77) (interaction *p* value = 0.005). Therefore, all required factors for A-1, −2, and −3 were confirmed to be met and reassessed to step 2.

We next evaluated the credibility assessment. The analysis at a cutoff of 1% was a post hoc analysis performed at clinical request; Design-1 was therefore evaluated as “No.” In addition, both Design-2 and Analysis-1 were evaluated “No” because PD-L1 evaluation was not mandatory, the analysis was limited to the 29.2% of patients who could be evaluated, and PD-L1 was neither a stratification factor nor adjusted for prognostic factors. Furthermore, another efficacy endpoint, PFS, was 0.73 (95% CI: 0.48–1.11) and 0.46 (95% CI: 0.33–0.64) for PD-L1 < 1% and PD-L1 ≥ 1%, respectively, indicating inconsistency in the HR assessment of OS and PFS in PD-L1 < 1%; thus, Analysis-2 was also rated “No.” Because the PACIFIC trial was the only trial at this time to investigate and confirm the efficacy of ICIs for locally advanced NSCLC, it cannot be compared with other independent trials; Context-1 was therefore evaluated as “NE.” Because many basic studies have shown that tumor PD-L1 expression is correlated with local immune response in tumors, suggesting that ICIs might have limited efficacy when PD-L1 is < 1%, Context-2 was rated “Yes.” Therefore, the credibility assessment was evaluated as moderate (+ 1).

Finally, the clinical assessment was conducted. Although CQs based on the PACIFIC trial have already been established in the JLCS guidelines, at this point, CQ recommendations were not divided on the basis of PD-L1 expression. Therefore, the indirectness of CQ was set to High (0). However, PD-L1 testing can be easily performed in daily practice, and a PD-L1 cutoff of less than 1% is a criterion relevant to treatment decision making, even in advanced-stage cases. Therefore, the clinical impact is High (+ 1). Although CQs based on the PACIFIC trial have already been established in the Japanese guidelines for lung cancer treatment, at this point, CQ recommendations were not divided on the basis of PD-L1 expression. Therefore, the risk evaluation was set at High (+ 1). The total point for the final step was three, and the final evaluation was Partially applicable.

[Case study 2]: Tumor PD-L1 expression subgroups in the IMpower010 trial (Supplementary Table 2 and Fig. 2).

The IMpower010 trial was a phase III study to evaluate the efficacy of atezolizumab after adjuvant chemotherapy in completely resected NSCLC [30]. In a prior PD-L1 test using resected specimens in this trial, we examined whether a subgroup with a cutoff of 50% positive tumor cell (TC) should be adopted into our guidelines. The primary endpoint, DFS, was significantly prolonged with atezolizumab in stage II–IIIA patients with PD-L1 TC ≥ 1%. However, in the subgroup analysis of DFS, the HR was 0.87 (95% CI: 0.60–1.26) for PD-L1 TC 1–49% and 0.43 (95% CI: 0.27–0.68) for PD-L1 TC ≥ 50%, indicating a difference in efficacy. In addition, a subgroup analysis based on PD-L1 in the updated OS report showed a HR of 0.95 (95% CI: 0.59–1.54) for PD-L1 TC 1–49%, which was inferior to the HR of 0.43 (95% CI: 0.24–0.78) for PD-L1 TC ≥ 50% (interaction *p* value = 0.041) [31]. Although these PD-L1 results were evaluated by staining with SP263 antibody, which was different from the pre-specified SP142 antibody, the analysis was considered less arbitrary because of the history of antibody change for versatility in real clinical settings. On the basis of the above, all the mandatory items of A-1, −2, and −3 were confirmed to be satisfied and the analysis was re-evaluated to step 2.

Next, the credibility assessment was evaluated. PD-L1 in this trial was a pre-specified analysis, and Design-1 was rated as “Yes.” PD-L1 was one of the stratification factors; however, the possibility of an imbalance between groups could not be ruled out because of the aforementioned change in the PD-L1 antibody; Design-2 was therefore rated “No.” By contrast, Analysis-1 was rated “Yes” because 97% of patients underwent PD-L1 analysis using SP263 and only 3% were excluded. The same efficacy tendency was observed for DFS and OS; therefore, Analysis-2 was rated"Yes."In assessing the reproducibility between trials, the PEARLS/KEYNOTE-091 trial—a phase III study that evaluated the efficacy of pembrolizumab as adjuvant therapy in completely resected NSCLC—is instructive [32]. This previous study found a DFS-HR of 0.67 (95% CI: 0.48–0.92) for PD-L1 TPS 1–49% and a DFS-HR of 0.82 (95% CI: 0.57–1.18) for PD-L1 TPS ≥ 50%, which was different from the IMpower010 trial. The PEARLS results were rather the opposite of conventional results; however, the reasons for this were not clear because the underlying background and results in advanced NSCLC showed that ICI intervention was more favorable in patients with greater tumor PD-L1 expression. Therefore, Context-1 was rated “No” and Context-2 was rated “Yes.” As a result, the credibility assessment was evaluated Very high (+ 3).

Finally, the clinical assessment was conducted. Atezolizumab was the first ICI shown to be effective as adjuvant therapy, and the CQ itself was a new item to be created. Therefore, the Indirectness of CQ was NE. As with the PACIFIC trial, the clinical impact of the results is considered to be high; therefore, the Clinical impact is rated High (+ 1). Among early-stage NSCLC patients, a certain percentage are expected to be cured without treatment after surgery, and interventions in this population must balance the benefits and harms. The potential for treatment-related death must be considered, as well as the risk of immune-related adverse events from ICI, such as adrenal insufficiency or type 1 diabetes, which may require permanent hormone replacement or supportive care. Therefore, the risk evaluation was set at High (+ 1). The total points for the final step was five, and the final evaluation was applicable, indicating that this CQ should be included as a new CQ in our guidelines.

## Discussion

We developed an evaluation algorithm of subgroup analysis for clinical practice guidelines and verified its availability by applying it to specific clinical trials in the field of lung cancer treatment. This algorithm incorporates clinical significance in addition to conventional statistical evaluation in subgroup analysis. This factor is important in introducing various types of evidence into clinical practice. We speculate that this evaluation procedure can be adapted as an evaluation criterion for subgroup analysis of clinical trials not only in lung cancer but also in a wide range of carcinomas.

Limitations include the arbitrariness of the decision/evaluation in some respects, and the lack of a safety assessment reflected in the evaluation algorithm. The safety may be a critical item worthy of evaluation in elderly populations and in certain racial groups. However, most subgroup analyses are based only on the efficacy of treatment; the literature contains few studies based on safety [17]. In the present study, we focused mainly on “efficacy” as an evaluation target. In addition, we developed this algorithm primarily to determine whether the results of a subgroup analysis from a single phase III trial should be applied to guidelines. The algorithm does not consider situations where conflicting results are obtained from multiple phase III trials simultaneously. We consider this issue a topic that should be addressed as the validation process progresses.

We have developed a new evaluation algorithm that incorporates clinical significance. We hope that this algorithm will be useful for evaluating appropriate subgroup analyses in clinical practice guidelines.

## Supplementary Information

Below is the link to the electronic supplementary material.

Supplementary file1 (DOCX 144 KB)
