# Supplementary material for: How to report and discuss subgroup analyses in clinical practice guidelines? Evaluation procedure of the clinical and statistical relevancy
Source: Int J Clin Oncol. 2025 May 11;30(7):1259–67. doi: 10.1007/s10147-025-02774-6 (PMC12187882; doi:10.1007/s10147-025-02774-6)

**Supplementary Table 1. Evaluation of the PD-L1 subgroups in the PACIFIC trial**

| **A. Required factors** | | | | | | |
| --- | --- | --- | --- | --- | --- | --- |
| 1. Does the primary outcome of the study show the meaningful benefit based on the intention-to-treat analysis? 2. Was the subgroup variable a pre-randomized factor? 3. Was there a meaningful difference between the subgroups in the interaction test?   (*p* < 0.05: surely; 0.05 ≤ *p* < 0.15: plausible ) | | | | | | Yes  Yes  Yes  *p* = 0.005 |
| **B. Credibility Assessment** | | | | | | |
| *Design* | 1. Was the subgroup hypothesis predetermined?  2. Was the subgroup factor a stratified allocation factor or adjusted for important prognostic factors? | | | | | No  No |
| *Analysis* | 1. Was the subgroup analysis performed on the basis of Intention-to-treat?  2. Was the subgroup analysis consistent with the relevant outcomes in this trial? | | | | | No  No |
| *Context* | 1. Was the subgroup analysis consistent with other independent clinical trials?  2. Was there a basic research context that could explain the subgroup effects obtained? | | | | | NE  Yes |
| Very high (+3) | | | High (+2) | Moderate (+1) | Low (0) | |
| **C. Clinical Assessment** | | | | | | |
| *Indirectness of CQ* | | Is the subgroup non-directive to the assumed clinical question? | | | High (0) | |
| *Clinical impact* | | Does the subgroup have a greater clinical impact on treatment decisions? | | | High (+1) | |
| *Risk evaluation* | | Does the subgroup influence treatment decisions due to risk of harm? | | | High (+1) | |
| **Final Decision** | |  | Applicable (≥4) | Partially applicable (2–3) | Not applicable (≤1) | |

**Supplementary Table 2. Evaluation of the PD-L1 subgroups in the IMpower010 trial**

| **A. Required Factors** | | | | | | |
| --- | --- | --- | --- | --- | --- | --- |
| 1. Does the primary outcome of the study show the meaningful benefit based on the intention-to-treat analysis? 2. Was the subgroup variable a pre-randomized factor? 3. Was there a meaningful difference between the subgroups in the interaction test?   (*p* < 0.05: surely; 0.05 ≤ *p* < 0.15: plausible ) | | | | | | Yes  Yes  Yes  *p* = 0.041 |
| **B. Credibility Assessment** | | | | | | |
| *Design* | 1. Was the subgroup hypothesis predetermined?  2. Was the subgroup factor a stratified allocation factor or adjusted for important prognostic factors? | | | | | Yes  No |
| *Analysis* | 1. Was the subgroup analysis performed on the basis of intention-to-treat?  2. Was the subgroup analysis consistent with the relevant outcomes in this trial? | | | | | Yes  Yes |
| *Context* | 1. Was the subgroup analysis consistent with other independent clinical trials?  2. Was there a basic research context that could explain the subgroup effects obtained? | | | | | No  Yes |
| Very high (+3) | | | High (+2) | Moderate (+1) | Low (0) | |
| **C. Clinical Assessment** | | | | | | |
| *Indirectness of CQ* | | Is the subgroup non-directive to the assumed clinical question? | | | Not evaluable | |
| *Clinical impact* | | Does the subgroup have a greater clinical impact on treatment decisions? | | | High (+1) | |
| *Risk evaluation* | | Does the subgroup influence treatment decisions due to risk of harm? | | | High (+1) | |
| **Final Decision** | |  | Applicable (≥4) | Partially applicable (2–3) | Not applicable (≤1) | |

Supplementary Figure 1. Interaction test of the PD-L1 subgroups in the PACIFIC trial


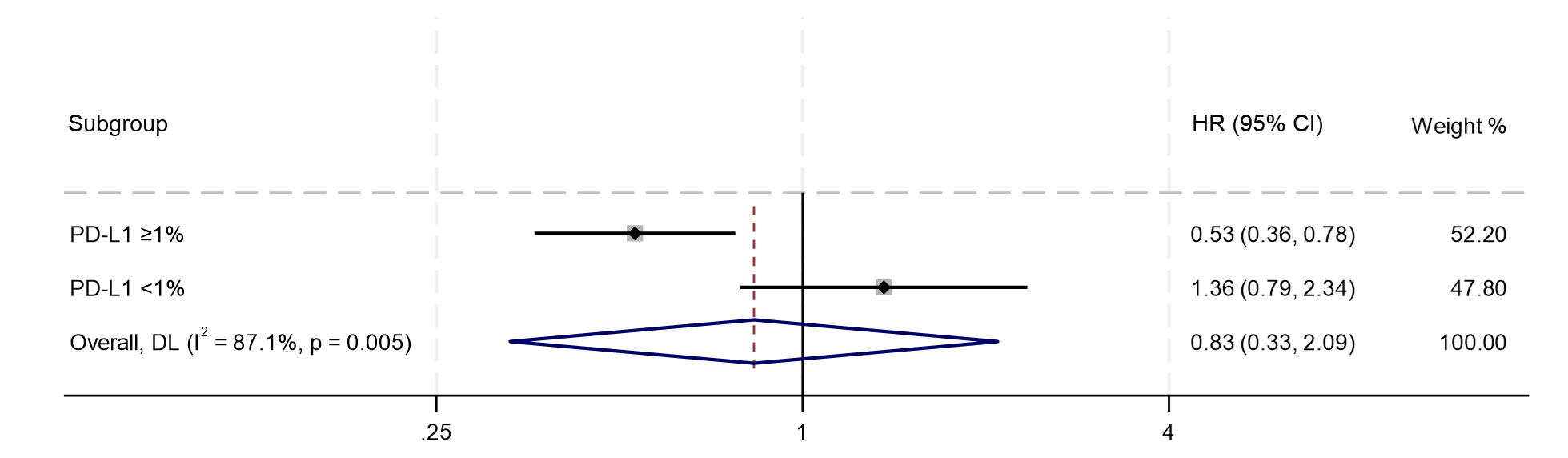


Supplementary Figure 2. Interaction test of the PD-L1 subgroups in the IMpower010 trial


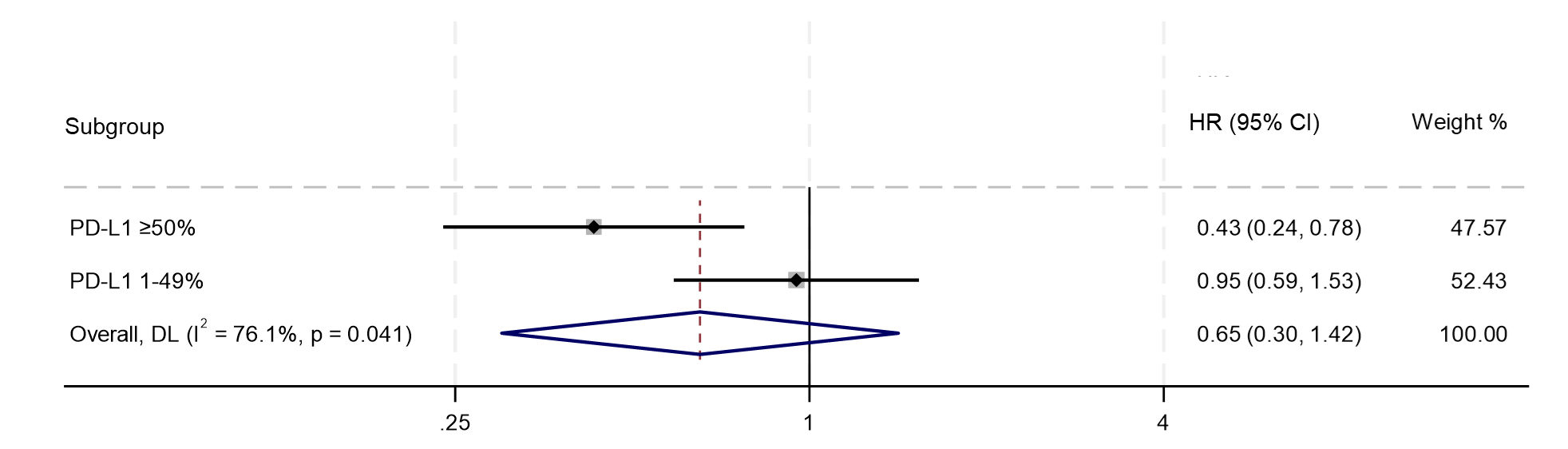

Supplement: Supplementary file 1 — Supplementary file1 (DOCX 144 KB) [file 10147_2025_2774_MOESM1_ESM.docx]
